# Applying an Exposome-Wide (ExWAS) Approach to Cancer Research

**DOI:** 10.3389/fonc.2018.00313

**Published:** 2018-08-27

**Authors:** Paul D. Juarez, Patricia Matthews-Juarez

**Affiliations:** Family and Community Medicine, Meharry Medical College, Nashville, TN, United States

**Keywords:** cancer, exposome, natural, built, social, environment, systems, pathways

## Abstract

Traditional research approaches, including genome-wide association studies (GWAS), epigenome-wide association studies (EWAS) and Gene × Environment (G × E) studies are limited in their ability to handle the multiplicity of chemical and non-chemical toxicants to which people are exposed in the real world, over their life course, their impact on epigenomics and other biological systems, and their relationship to cancer onset, progression, and outcomes. Exposome-wide association study (ExWAS) provides a new approach for conceptualizing the roles and relationships of multiple chemical and non-chemical exposures in the etiology and progression of cancer at key developmental periods, over the life course, and across generations. ExWAS challenges us to consider the influence of both internal and external environment, chemical and non-chemical stressors, risk and protective factors, and spatial and temporal dimensions of exposures in our models of cancer incidence, outcomes, and disparities. Applying an ExWAS approach to cancer and cancer disparities research supports robust computational models and methods that will allow for analysis of the dynamic and complex interactions between genetics, epigenetics, and exposomics factors. In the coming months, we will spatially and temporally align environmental exposures with SCCS participant data from time of enrollment forward to move us closer to identifying complete exposure pathways that lead to cancer. In the future, we hope to link external sources of exposure to biomarkers of exposure, biomarkers of disease, disease phenotypes, and population level disparities.

## Introduction

Traditional research approaches are limited in their ability to handle the multiplicity of chemical and non-chemical toxicants to which people are exposed in the real world and over their life course. Cancer and cancer disparities research have been limited by these traditional methods.

A litany of chemical and non-chemical exposures emanating in the external environment have been linked to cancer and cancer disparities, including contaminants found in the air, water, and land and/or soil, materials used in houses and office buildings, psycho-social stressors, individual behavior, and public policies, among others (1, 2). Traditional research methods and analytics, however, have yielded little progress over the past four decades in identifying either the causes of cancer or of racial/ethnic cancer disparities in communities which shoulder the greatest burden. This commentary posits that cancer and cancer health disparities research may benefit tremendously by using an exposome-wide association study (ExWAS) framework previously described as the “public health exposome” and pioneered by Juarez et al. (3). The internal environment of the exposome is discussed in other papers and will not be addressed in much detail (4–6).

The exposome, first introduced in 2005 by Wild (7) addresses the limitations of traditional research approaches by applying a systems approach for considering the relationships between external exposures and internal genetic, epigenetic, and exposomic factors in the onset and progression of disease (8). Exposome-wide association study (ExWAS) provides a new approach for conceptualizing the roles and relationships of multiple chemical and non-chemical exposures in the etiology and progression of cancer at key developmental periods, over the life course, and across generations. ExWAS supports both data driven and hypothesis driven approaches and the application of combinatorial analytics to population health and health disparities making it possible to systematically explore the complex relationships between external and internal environments and health over the life course. Langston et al. (9) have demonstrated the applicability of scalable combinatorial methods, which are algorithmically sophisticated, highly automated and mathematically abstract, from genome science research, to the study of population health. Unlike standard techniques which can scrutinize at most a handful of parameters for obvious dependencies, combinatorial methods are able to extract latent signal from large heterogeneous data sets with only modest correlations spread across an entire spectrum of available variables.

Applying an ExWAS approach to cancer and cancer disparities research supports robust computational models and methods that will allow for analysis of the dynamic and complex interactions between genetics, epigenetics, and exposomics factors (3). This approach which is based in systems theory and is adaptable to real world conditions, portends significant improvements in our understanding of the mechanisms and pathways that lead to cancer and cancer disparities, and builds on and provides significant advantages over current genome-wide association studies (GWAS), Gene × Environment (G × E) efforts, and epigenome-wide association studies (EWAS). While GWAS alone continues to provide a valuable approach to understanding some cancers that are primarily genetic in origin, its major limitation is its singular focus on genetic variants in individuals. Likewise, while G × E interaction studies offer important clues about the relationships between environmental exposures and genetic mutations, they have been limited by a focus on how a handful of environmental exposures are associated with genetic differences. In addition, they have been difficult to replicate and are readily included in an ExWAS approach. While EWAS, with a focus on the role of epigenetic marks, makes a valuable contribution to our understanding of underlying mechanisms and pathways through which environment may affect cancer and other chronic diseases, it can be easily incorporated within a more comprehensive ExWAS approach. A major innovation of an ExWAS approach is that it supports the inclusion of both external environmental exposures and internal biological mechanisms and pathways (genetic, epigenetic, omics), and their effects on health outcomes. Understanding sources, amount, duration, and age of carcinogenic exposures in the environment as well as protective factors, such as social support, are important for understanding cancer risk, translational elements, and resilience.

Furthermore, an ExWAS approach applies a dynamic, trans-disciplinary, exposure science frame and mechanistic approach for understanding cancer risk within a real-world environment of multiple exposures, cumulative, antagonistic, and synergistic interactions, changing risk status, susceptible periods of development, and transgenerational transmission of disease, while accounting for individual differences in genetic, epigenetic, and omics profiles (10). Applying an ExWAS approach to cancer moves our understanding of how to conceptualize incidence, outcomes, and disparities beyond genome-wide (GWAS) and epigenome-wide (EWAS) approaches, to capture the complexity of multiple and continuous environmental exposures encountered in the real world. The building blocks of the external environmental exposome include a multi-dimensional interactome (11) that includes mechanisms, pathways, and the interactions between internal (i.e., genetics, epigenetics, metabolomics, transcriptomic, proteomics, etc.) and external environments (natural, built, social, and policy) (12). Other research groups that have adapted an exposome approach include the European Human Early-Life Exposome (HELIX): Project (13); the Hercules Exposome Research Center at Emory University (Niedwiecki and Miller, (14), and federal research institutes and agencies including NIEHS (Exposure Biology and the Exposome, accessed on 6/24/2018 at: https://www.niehs.nih.gov/research/supported/exposure/bio/index.cfm); EPA (Discovering Environmental Causes of Disease: From Exposure Biology to the Exposome, accessed on 6/4/2018 at: https://www.epa.gov/chemical-research/discovering-environmental-causes-disease-exposure-biology-exposome); and NIMHD (NIMHD Science Visioning. Accessed pm 6/24/2018 at: https://www.nimhd.nih.gov/docs/NIMHD_RFI_Summary_508.pdf).

## External environment taxonomy

Perhaps the greatest single challenge to an ExWAS approach is the need for development of a robust ontology that will allow for the systematic classification of exposures from the external environment and their hierarchical relationships. In order to obtain widespread use, this effort, may require the development of a standards development organization, such as SNOMED International to ensure the development of a consistent means to index, retrieve, aggregate, analyze, and interpret findings (SNOMED International, accessed on 6 24 2018 at: https://www.snomed.org/). SNOMED CT was developed as a systematic ontology of terms that can be used in health informatics anywhere in the world to ensure systematic recording of clinical data to support patient care. Other challenges of using an ExWAS approach include the inconsistency in spatial and temporal measurements, limited knowledge of the “universe” of external environmental exposures that may have a direct or indirect impact on health outcomes and disparities, and the lack of an informatics infrastructure to support ongoing data collection, curation, and updating efforts.

Juarez (15) operationalized the public health exposome by creating a taxonomy of external environmental exposures and curated a database, that to date, includes over 30,000 attributes of exposure across the natural, built, social, and policy environments which can be used to assess exposure risk, protective factors, and interactive effects. The structure of the public health exposome database includes annual, county level measures for all 3,142 US counties and county-equivalents for up to 30 years. It also includes daily, 3-km, gridded measures of PM2.5 for 12 southeastern states developed from a regional regression model and a spatial surfacing algorithm derived from MODIS satellite measures and ground monitoring station data (16). Juarez et al. has promulgated and published “proof of concept” articles using the curated database that demonstrated that ExWAS approach can examine large data sets and produce results that can be duplicated and verified (17–20).

The public health exposome database recently was linked to data from the Southern Community Cohort Study (SCCS), (21) a cohort of 85,000 adults recruited primarily through community health centers in 12 southeastern states under a recently received EPA STAR grant. The SCCS data include three waves of survey data which include personal characteristics, activities/behaviors, health history, administrative Medicaid and Medicare data, and Social Security Death Index files. The SCCS data base also is linked to state cancer registries and has biospecimens (blood, urine, and buccal cells) available for most participants. The residential histories of SCCS participants have been geocoded allowing for spatial and temporal alignment of external and internal exposure and health data. The results of this EPA STAR grant are expected to demonstrate the effectiveness of ExWAS in assessing life-long effects of chemical and non-chemical environmental exposures to cancer across the life course by providing a means to align, both spatially and temporally, environmental exposures with the onset and progression of disease across the life course. In the coming months, we will spatially and temporally align environmental exposures with SCCS participant data from time of enrollment forward to move us closer to identifying complete exposure pathways that lead to cancer. In the future, we hope to link external sources of exposure to biomarkers of exposure, biomarkers of disease, disease phenotypes, and population level disparities.

In conclusion, ExWAS challenges us to consider the influence of both internal (e.g., biological mechanisms and pathways) and external environment, chemical and non-chemical stressors, risk and protective factors, and spatial and temporal dimensions of exposures in our models of cancer incidence, outcomes, and disparities (22, 23). ExWAS supports a data driven approach that leverages existing, spatially and temporally coded, publicly available data and has implications for the development, targeting, and evaluation of public health interventions, programs, and policies. ExWAS portends new discoveries about the effects of environmental exposures on the onset and progression of cancer; the effects of exposures at key developmental periods, across the life course, and trans-generationally by spatially and temporally linking external, internal, and health outcomes data; previously unidentified risk and protective factors that can be uncovered by applying data driven computational methods; and the effects of cumulative and interactive exposures. We suggest that use of an ExWAS approach and the “Public Health Exposome” framework, will lead to new discoveries in research that will contribute to our understanding of the causes of cancer in general and cancer disparities among racial/ethnic and disadvantaged groups, in particular.

## Author contributions

PJ was responsible for drafting the paper. PM-J was responsible for refining and editing the paper.

### Conflict of interest statement

The authors declare that the research was conducted in the absence of any commercial or financial relationships that could be construed as a potential conflict of interest.
